# Neutrophil Percentage-to-Albumin Ratio as a Prognostic Marker in Pneumonia Patients Aged 80 and Above in Intensive Care

**DOI:** 10.3390/jcm14093033

**Published:** 2025-04-28

**Authors:** Maside Ari, Aslı Haykir Solay, Tarkan Ozdemir, Murat Yildiz, Oral Mentes, Omer Faruk Tuten, Husra Tetik Manav, Deniz Celik, Melek Doganci, Guler Eraslan Doganay, Emrah Ari, Eren Usul

**Affiliations:** 1 Department of Pulmonology, Ankara Ataturk Sanatorium Training and Research Hospital, 06290 Ankara, Türkiye; drmuratyildiz85@gmail.com (M.Y.); hsratetik@gmail.com (H.T.M.); 2 Department of Infectious Diseases and Microbiology, Ankara Etlik City Hospital, 06170 Ankara, Türkiye; aahaykir@hotmail.com; 3 Department of Pulmonology, Konya Farabi Hospital, 42090 Konya, Türkiye; tarkanozdemir78@gmail.com; 4 Clinic of Intensive Care Unit, Ankara Gulhane Training and Research Hospital, 06010 Ankara, Türkiye; omentes@live.com; 5 Department of Pulmonology, Ankara University Health Practise and Research Hospitals, 06050 Ankara, Türkiye; omertuten@gmail.com; 6 Department of Pulmonology, Alanya Alaaddin Keykubat University Education and Research Hospital, 07450 Antalya, Türkiye; drdenizcelik@hotmail.com; 7 Clinic of Anesthesiology and Reanimation, Ankara Ataturk Sanatorium Training and Research Hospital, 06290 Ankara, Türkiye; melekdidik@hotmail.com (M.D.); gulerdoganay@hotmail.com.tr (G.E.D.); 8 Department of Emergency Medicine, Mamak Public Hospital, 06270 Ankara, Türkiye; dremrahari25@gmail.com; 9 Department of Emergency Medicine, Ankara Etlik City Hospital, 06170 Ankara, Türkiye; usuleren7@hotmail.com

**Keywords:** oldest old, mortality, NPAR, pneumonia, intensive care

## Abstract

**Background/Objectives:** In recent years, inflammatory markers have been increasingly utilized to predict disease prognosis. The neutrophil percentage-to-albumin ratio (NPAR) has emerged as a novel biomarker reflecting inflammation and systemic response. This study was conducted to evaluate the prognostic value of NPAR in pneumonia patients aged 80 years and older hospitalized in intensive care. **Methods:** Patients aged 80 years and older who were followed up in the intensive care unit with a diagnosis of pneumonia between 1 October 2022, and 31 May 2024, were retrospectively reviewed. Demographic characteristics, laboratory data, disease severity scores (APACHE II, SOFA), intensive care interventions, and variables associated with mortality were analyzed. NPAR was calculated by dividing the neutrophil percentage by the serum albumin level. The prognostic value of NPAR was assessed using Kaplan–Meier survival analysis, receiver operating characteristic (ROC) curve analysis, and Cox regression analysis. **Results:** A total of 135 patients were included in the study. Patients with NPAR > 0.286 had significantly higher SOFA (*p* = 0.002) and APACHE II (*p* = 0.007) scores. The high NPAR group was at significantly greater risk for requiring invasive mechanical ventilation (*p* = 0.003), vasopressor support (*p* = 0.042), and developing sepsis (*p* = 0.035). Elevated NPAR was strongly associated with mortality (*p* < 0.001) and was identified as an independent predictor of mortality in the Cox regression analysis (HR = 2.488, 95% CI: 1.167–5.302, *p* = 0.018). **Conclusions:** NPAR may serve as an effective biomarker for predicting disease severity and mortality risk in pneumonia patients aged 80 years and older. Due to its simplicity and accessibility, it can be considered a practical parameter for integration into clinical practice. However, large-scale, multicenter, and prospective studies are needed to validate these findings.

## 1. Introduction

Individuals aged 80 years and older constitute a distinct age group referred to as the “oldest old” [1]. With the global increase in life expectancy, this population is projected to triple by the year 2050 [2]. The growing elderly population places a significant burden on healthcare systems by increasing the demand for medical care. Especially in oldest old individuals, the high burden of comorbid diseases, weakened immune systems and decreased functional capacity increase the susceptibility to infections and increase the risk of complications. Pneumonia in oldest old patients is often associated with more severe clinical presentations and frequently necessitates intensive care admission in the presence of any infection [3]. Moreover, the incidence of pneumonia cases requiring intensive care among this age group has been reported to be rising [4]. Therefore, accurate prediction of disease prognosis in oldest old individuals is of great importance to ensure early and appropriate interventions.

Neutrophils are key components of the innate immune system and represent the first line of defense against infections by mounting a rapid response to invading pathogens. During infectious and inflammatory processes, both the count and percentage of neutrophils increase rapidly, reflecting the magnitude of the systemic inflammatory response. Therefore, the neutrophil percentage is considered a biological indicator of infection severity and the level of inflammation [5]. Although albumin is commonly associated with nutritional status, it also functions as a negative acute-phase reactant. In the presence of systemic inflammation, hepatic synthesis of albumin decreases, capillary permeability increases, and albumin shifts into the extravascular space, resulting in reduced serum levels. This decline may reflect not only malnutrition but also the severity of the infectious or inflammatory process [6]. Due to these characteristics, albumin serves as an important parameter in prognostic assessment of infectious diseases.

In recent years, there has been a growing focus on the use of simple, rapid, and widely accessible biomarkers to evaluate inflammatory states. In this context, the neutrophil percentage-to-albumin ratio (NPAR) has emerged as a promising parameter [7]. NPAR simultaneously reflects the acute inflammatory response mediated by neutrophils and the systemic inflammatory and nutritional status represented by albumin levels [8]. Thus, it functions as a dual-purpose biomarker, indicating both infection severity and the patient’s physiological reserve. Previous studies have demonstrated that NPAR is associated with mortality and adverse clinical outcomes in various conditions such as acute kidney injury, cardiovascular diseases, stroke, and sepsis [9,10,11,12]. However, the majority of these studies have focused on the general adult population or relatively younger patient groups. Data evaluating the relationship between NPAR and clinical outcomes in the very elderly population (aged ≥ 80 years) remain scarce. In older adults, reduced physiological reserve, immunosenescence, and the presence of multiple comorbidities contribute to a variable response to infections, potentially affecting the prognostic utility of biomarkers.

In this study, we aimed to investigate the prognostic value of NPAR in elderly patients with pneumonia and to evaluate its association with disease severity and clinical outcomes. We believe that the findings of this study may contribute to clinical decision-making in the management of older patients admitted to the intensive care unit.

## 2. Materials and Methods

**Figure 1:**
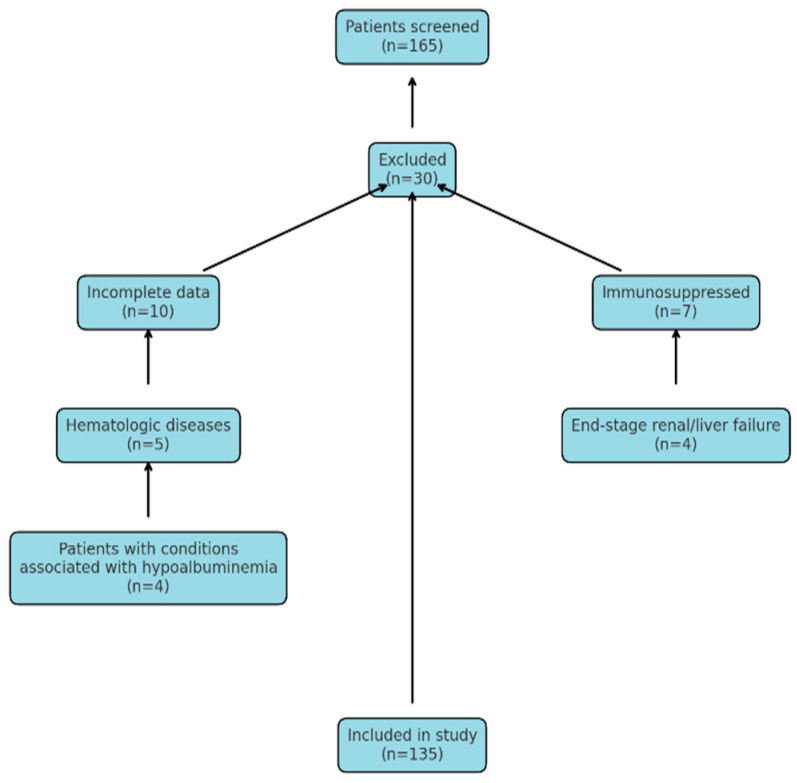
Flowchart of patients included in and excluded from this study.

This study included patients aged 80 years and older who were followed in the intensive care units of Ankara Atatürk Sanatorium Training and Research Hospital between 1 October 2022, and 31 May 2024. Data from patients diagnosed with community-acquired pneumonia were retrospectively reviewed using the hospital information system and patient medical records. Figure 1 shows a flowchart detailing the patients included in and excluded from this study.

This study was approved by the Ankara Atatürk Sanatorium Training and Research Hospital Clinical Research Ethics Committee with decision number 2839 dated 16 July 2024 and was conducted in accordance with the ethical principles stated in the Declaration of Helsinki.

The diagnosis of pneumonia was established based on the presence of the following three criteria after excluding alternative diagnoses:

1. Symptoms of lower respiratory tract infection: Fever (>38 °C), cough, purulent sputum, or a change in the character of respiratory secretions.
2. Radiographic findings consistent with pneumonia: Newly developed infiltrates on chest radiography or thoracic computed tomography.
3. Laboratory findings suggestive of infection: Leukocytosis, leukopenia, or elevated acute phase reactants.

### 2.1. Exclusion Criteria: Patients Who Were Not Included in the Study Were Identified Based on the Following Exclusion Criteria

Incomplete or insufficient patient data: Missing essential clinical, laboratory, or radiological data in the hospital information system or patient records.

Primary diagnoses other than pneumonia: Patients whose primary diagnosis was not pneumonia and who had alternative conditions that could mimic lower respiratory tract infections (e.g., pulmonary embolism, pulmonary edema due to congestive heart failure, interstitial lung diseases, pulmonary infiltrates due to malignancy).

Immunosuppressed patients: Patients with a history of chemotherapy, long-term corticosteroid use (>20 mg/day prednisone equivalent), immunosuppressive therapy, or solid organ/bone marrow transplantation.

Severe hematologic diseases: Patients with significant immune system impairment due to leukemia, lymphoma, or severe bone marrow failure.

End-stage renal or liver failure: Patients with end-stage chronic kidney disease (stage 5 requiring dialysis) or cirrhosis classified as Child–Pugh class C.

Diseases associated with hypoalbuminemia: Patients diagnosed with conditions that could cause hypoalbuminemia, such as chronic liver diseases or nephrotic syndrome.

### 2.2. Data Collection and Evaluation

Patients’ comorbidities were recorded, and the most common comorbidities were identified. The impact of these comorbidities on mortality was also analyzed. Demographic data, clinical findings, complete blood count and biochemical parameters obtained within the first 24 h of intensive care unit (ICU) admission, acute phase reactants, imaging findings, administered treatments, need for respiratory and vasopressor support, requirement for renal replacement therapy, and patient outcomes were collected through the hospital information system and patient files.

The primary outcome of the study was all-cause mortality occurring within 30 days during hospitalization. Patients were followed until hospital discharge or death within the 30-day period.

The neutrophil percentage-to-albumin ratio (NPAR) was calculated using laboratory data obtained within the first 24 h of ICU admission and its association with clinical outcomes was evaluated.

Neutrophil percentage was measured using a Mindray BC-6800 automated hematology analyzer (Shenzhen Mindray Bio-medical Electronics Co., Ltd., Shenzhen, China) and recorded as a percentage. Serum albumin levels were measured in g/L using a Beckman Coulter AU680 chemistry analyzer (Beckman Coulter Inc., Brea, CA, USA). NPAR was calculated by dividing the neutrophil percentage by the serum albumin level.

### 2.3. Assessment of Sepsis and Disease Severity

The diagnosis of sepsis was made according to the international Sepsis-3 consensus criteria. Patients diagnosed with pneumonia and found to have a Sepsis-Related Organ Failure Assessment (SOFA) score ≥ 2 at the time of ICU admission were considered to have sepsis [13,14]. Patients who required vasopressor support to maintain a mean arterial pressure of ≥65 mmHg despite adequate fluid resuscitation were classified as having septic shock.

To objectively assess disease severity, the Acute Physiology and Chronic Health Evaluation II (APACHE II) score—commonly used in intensive care settings—was also calculated at the time of admission [15].

### 2.4. Calculation of SpO_2_/FiO_2_

In this study, the SpO_2_/FiO_2_ ratio was calculated to evaluate the patients’ oxygenation status. SpO_2_ values were obtained using a standard pulse oximeter, and the FiO_2_ level was recorded based on the concentration of inspired oxygen. For patients receiving supplemental oxygen, FiO_2_ was estimated according to the oxygen flow rate and the method of oxygen delivery. The SpO_2_/FiO_2_ ratio was calculated by dividing the SpO_2_ value by the FiO_2_ value. This calculation was performed within the first 24 h following admission to the intensive care unit. This ratio was used to classify the hypoxemic status of patients.

### 2.5. Patient Selection

In this study, all participants were admitted to the ICU either directly from the Emergency Department or following initial evaluation and short-term monitoring in the Department of Pulmonology. To ensure homogeneity of the study cohort and minimize variability related to the timing of clinical deterioration, only patients whose total time from initial hospital admission to ICU transfer was less than 24 h were included. This inclusion criterion was intended to capture cases of early critical illness and avoid confounding from complications developing during prolonged general ward stays.

### 2.6. Statistical Analysis

Statistical analyses were performed using SPSS version 27 (Statistical Package for the Social Sciences). The normality of distribution for continuous variables was assessed using the Kolmogorov–Smirnov test. Variables with normal distribution were expressed as mean ± standard deviation (Mean ± SD), while non-normally distributed variables were expressed as median and interquartile range (IQR, 25th–75th percentiles). Appropriate parametric or non-parametric tests were used to compare differences between groups. For comparisons between two independent groups, the t-test or Mann–Whitney U test was applied for continuous variables. The chi-square test (χ^2^) or Fisher’s exact test was used for categorical variables. The prognostic performance of NPAR in predicting mortality was evaluated using receiver operating characteristic (ROC) curve analysis. The area under the curve (AUC) was calculated for each variable, and optimal cutoff values were presented along with sensitivity and specificity. For survival analysis, Kaplan–Meier curves were generated, and differences between groups were assessed using the log-rank test. Cox regression analysis was used to identify factors associated with mortality. Initially, univariate Cox regression analysis was performed to identify candidate variables, and significant variables were then included in the multivariate model. Results of the model were reported as hazard ratios (HR) with 95% confidence intervals (CI). A *p*-value of <0.05 was considered statistically significant.

## 3. Results

A total of 135 patients were included in the study. In Table 1, demographic characteristics, clinical scores, laboratory parameters, and supportive treatment needs are compared between survivors and non-survivors. Among non-survivors, SOFA and APACHE II scores, as well as NPAR, procalcitonin, lactate, blood urea nitrogen, and creatinine levels were significantly higher, whereas albumin and platelet levels were lower. Furthermore, the need for invasive mechanical ventilation, hemodialysis, and vasopressor support was markedly higher in this group.

Patients were divided into two groups, NPAR ≤ 0.286 and NPAR > 0.286, based on the cut-off value of 0.286 determined as a result of ROC analysis to evaluate the prognostic value of NPAR.

When the clinical characteristics of the patients were compared between groups, disease severity markers were found to be higher and mortality rates significantly increased in the high NPAR group. SOFA (*p* = 0.002) and APACHE II (*p* = 0.007) scores were significantly higher in patients with elevated NPAR. The need for invasive mechanical ventilation (*p* = 0.003), vasopressor therapy (*p* = 0.042), and the incidence of sepsis (*p* = 0.035) were also significantly greater in the high NPAR group. Moreover, mortality was significantly higher in patients with elevated NPAR (*p* < 0.001) (Table 2).

When laboratory findings were compared according to NPAR levels, inflammatory and metabolic markers were found to be significantly elevated in the high NPAR group. Higher NPAR levels were associated with increased procalcitonin (*p* = 0.020) and lactate (*p* = 0.003) levels (Table 3).

**Figure 2:**
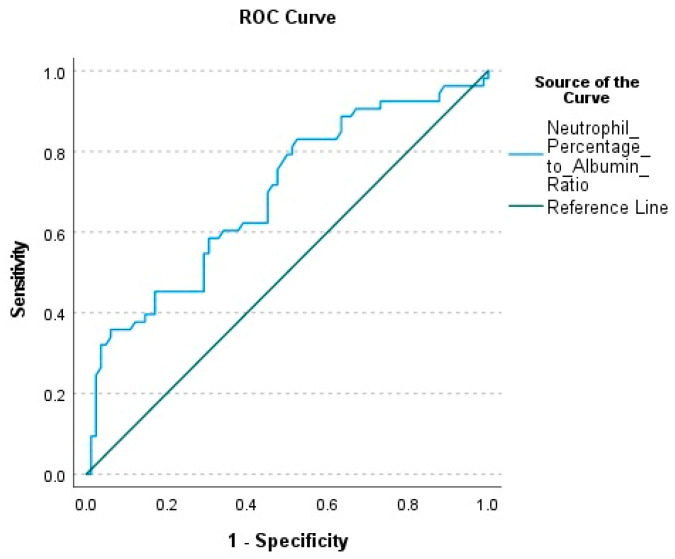
ROC Curve of Neutrophil Percentage-to-Albumin Ratio (NPAR) for Predicting Mortality.

NPAR was calculated as 0.291 (0.244–2.16) in survivors and 0.422 (0.298–3.092) in deceased patients. This difference between the groups was statistically significant (*p* < 0.001). The AUC value calculated to evaluate the mortality prediction power of NPAR was found to be 0.692. (*p* < 0.001). The optimal cut-off value was determined as 0.286, with a sensitivity of 83%, specificity of 47.6%, positive predictive value (PPV) of 50.6%, and negative predictive value (NPV) of 81.2% (Table 4) (Figure 2).

In the univariate Cox regression analysis, patients with NPAR > 0.286 had a significantly increased risk of mortality (HR = 3.318, 95% CI: 1.616–6.812, *p* = 0.001). Similarly, mortality was significantly increased in patients with high APACHE-II and SOFA scores. The need for renal replacement therapy and vasopressor support were also significantly related to mortality. The strongest association was observed with the requirement for invasive mechanical ventilation (IMV); patients who required IMV had an approximately 20-fold higher risk of mortality (HR = 20.297, 95% CI: 8.019–51.374, *p* < 0.001). In contrast, variables such as cardiovascular disease (*p* = 0.524), hypertension (*p* = 0.172), and the presence of comorbidities (*p* = 0.078) were not significantly associated with mortality.

In the multivariate analysis, an NPAR level > 0.286 was identified as an independent risk factor for mortality (HR = 2.488, 95% CI: 1.167–5.302, *p* = 0.018). The APACHE II score remained significantly associated with increased mortality risk (HR = 1.077, 95% CI: 1.013–1.147, *p* = 0.019), whereas the SOFA score was not found to be an independent predictor in the multivariate model (*p* = 0.156). The need for renal replacement therapy was also determined to be an independent predictor of mortality (HR = 1.969, 95% CI: 1.046–3.705, *p* = 0.036). The requirement for invasive mechanical ventilation remained the strongest independent risk factor (Table 5).

When comorbid conditions were evaluated, the rates of hypertension and cardiovascular disease were found to be higher in the low NPAR group (*p* < 0.001 and *p* = 0.007, respectively). However, when the association of comorbidities with mortality was assessed using Cox regression analysis, neither hypertension (*p* = 0.172) nor cardiovascular disease (*p* = 0.524) showed a statistically significant relationship.

**Figure 3:**
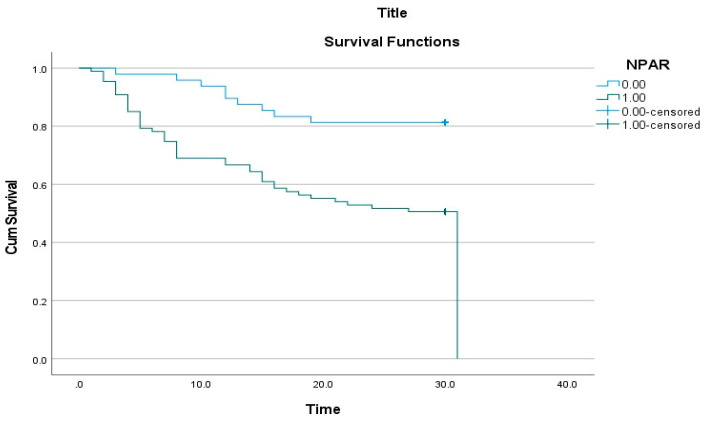
Kaplan–Meier Analysis of the Association Between Neutrophil Percentage-to-Albumin Ratio (NPAR) and Mortality.

According to the Kaplan–Meier survival analysis, the survival rate was significantly lower in the high NPAR group (Figure 3). The log-rank test revealed a statistically significant difference in survival times between the NPAR groups (*p* < 0.001).

## 4. Discussion

This study was conducted to evaluate the prognostic value of the neutrophil percentage-to-albumin ratio (NPAR) in pneumonia patients aged 80 years and older admitted to the intensive care unit. Our findings demonstrate that elevated NPAR levels are significantly associated with disease severity markers such as SOFA and APACHE II scores, and may be linked to worse clinical outcomes during the intensive care course. The results of the Kaplan–Meier survival analysis showed that higher NPAR levels were associated with significantly lower survival rates. Furthermore, in the multivariate Cox regression analysis, NPAR was identified as an independent predictor of mortality, and this association was found to be independent of other clinical variables such as comorbidities, disease severity, and organ failure. Elevated NPAR was also significantly associated with the need for invasive mechanical ventilation (IMV), vasopressor use, and the development of sepsis. This suggests that NPAR may also reflect critical clinical conditions such as hemodynamic instability and organ dysfunction. Based on these findings, NPAR—as a simple, rapid, and widely accessible laboratory parameter—may be considered a clinically useful biomarker for predicting disease severity and mortality risk in pneumonia patients aged 80 years and older. However, for a more comprehensive evaluation of this relationship, large-scale, multicenter prospective studies including different patient populations are needed.

In very elderly individuals, the immune response to infections is significantly influenced by the presence of comorbid conditions. Chronic diseases in this age group affect not only susceptibility to infections but also the severity of the clinical course and the need for intensive care. According to the literature, chronic pulmonary diseases, diabetes mellitus, cardiovascular diseases, and neurological disorders are among the most commonly reported comorbidities, and they have been associated with increased rates of sepsis and mortality [4,16]. In our study, at least one comorbid condition was present in 82.2% of the patients, with cardiovascular diseases and chronic obstructive pulmonary disease (COPD) being the most prevalent. Although the mortality rate was significantly higher among patients with comorbidities, multivariate regression analysis did not identify the presence of comorbidity as an independent predictor of mortality.

Interestingly, we observed that patients with higher NPAR values tended to have fewer comorbidities. This finding may be related to the prioritization of acute illness severity over chronic disease burden during ICU admission. Patients with fewer comorbidities but a more pronounced inflammatory response—and therefore a more severe clinical presentation—may have been more likely to be admitted to intensive care. This observation suggests that, in elderly patients, not only the presence of comorbidities but also biomarkers reflecting the degree of systemic inflammation may play a key role in patient management and risk stratification.

Serum albumin is a negative acute-phase reactant involved in inflammatory processes and exhibits antioxidant properties by interacting with bioactive lipid mediators, which are critical components of the immune response [17]. Malnutrition has previously been shown to be associated with poor clinical outcomes in patients with pneumonia [18]. It is well established that, with advancing age, nutritional deficiencies are directly related to immune system impairment. In our study, hypoalbuminemia, along with elevated NPAR, was significantly associated with increased mortality. This finding suggests that low albumin levels may influence pneumonia prognosis through mechanisms related to both inflammation and nutritional status. Therefore, in elderly patients with pneumonia, clinicians should consider not only nutritional status but also the underlying inflammatory state. When necessary, in addition to early nutritional support, interventions targeting the control of the inflammatory response should also be prioritized.

Neutrophils are key components of the systemic inflammatory response to infection and represent one of the most important cells of the innate immune system. Moreover, they are known to be closely associated with organ dysfunction in the setting of severe infections and sepsis [19]. In recent years, NPAR has been evaluated across various disease groups and has emerged as a promising prognostic biomarker. Elevated NPAR levels have been shown to be associated with mortality in patients with cerebrovascular diseases, cardiovascular diseases, and chronic obstructive pulmonary disease (COPD) [8,9,20]. In a study conducted on ICU patients diagnosed with sepsis, NPAR measured at admission was reported to be a significant predictor of 28-day mortality [21]. Although the prognostic role of NPAR has been explored in a range of clinical settings, the majority of these studies have focused on the general population or relatively younger patients. For instance, in the study by Hu et al. involving septic patients, the median age was approximately 65 years, whereas in our study, the median age was 86. Similarly, in the NHANES database analysis by Lan et al., which included a COPD population, the mean age was below 70 years [8]. While these studies have demonstrated a significant association between elevated NPAR and mortality, age-related changes such as variability in the inflammatory response, decreased serum albumin levels, and an increased burden of comorbidities may influence the prognostic performance of this parameter in older adults. Our study is among the few to evaluate the prognostic value of NPAR specifically in the very elderly population and demonstrates that NPAR is significantly associated with mortality in this age group. This finding supports the potential of NPAR as a simple and practical biomarker for early risk stratification in older patients.

A high NPAR value reflects an increased systemic inflammatory burden, resulting from an elevated neutrophil percentage, decreased serum albumin levels, or a combination of both. In patients with pneumonia, this condition simultaneously indicates a severe inflammatory response as well as compromised nutritional and physiological reserves. In elderly individuals, physiological changes such as immunosenescence, chronic low-grade inflammation, and malnutrition are common. These alterations impair immune defenses and negatively affect recovery from infections. Therefore, elevated NPAR levels in older adults may be directly associated with adverse clinical outcomes and increased mortality risk. In our study, NPAR demonstrated prognostic sensitivity comparable to that of the widely used SOFA score. Given that it is derived from only two routine laboratory parameters, NPAR may serve as a practical biomarker to support clinical decision-making in elderly patients with pneumonia.

Recent research suggests that NPAR may serve not only as a prognostic marker but also as a potential indicator for guiding therapeutic strategies. For instance, a study by Liu and Chien reported that elevated NPAR levels were significantly associated with non-alcoholic fatty liver disease and advanced liver fibrosis [7]. This finding highlights the ability of NPAR to reflect both systemic inflammation and nutritional deficits. Accordingly, early detection of elevated NPAR levels in patients with pneumonia may act as a clinical warning sign, prompting timely, targeted interventions. Specifically, early nutritional support to address hypoalbuminemia and anti-inflammatory strategies aimed at controlling neutrophil-mediated inflammation may improve outcomes in this vulnerable patient population.

According to the intensive care unit admission criteria established by the American Thoracic Society (ATS) and the Infectious Diseases Society of America (IDSA), the need for vasopressors and mechanical ventilation are considered major indicators in critically ill patients [22]. In our study, mortality was found to be higher in patients who required vasopressor support and invasive mechanical ventilation (IMV), and these factors were identified as independent predictors of mortality. Additionally, patients with elevated NPAR levels were significantly more likely to require vasopressor therapy and IMV. These findings suggest that NPAR may reflect not only inflammatory processes but also critical clinical conditions such as hemodynamic instability and respiratory failure.

The APACHE II and SOFA scores are widely used scoring systems for assessing disease severity and predicting mortality in critically ill patients [23,24,25]. In our study, the APACHE II score was found to be an independent prognostic predictor of mortality, whereas the SOFA score did not remain significant in the multivariate analysis. Additionally, increases in both APACHE II and SOFA scores were significantly associated with elevated NPAR levels. An NPAR level > 0.286 was shown to be an independent predictor of mortality. These findings suggest that NPAR may serve as a biomarker reflecting disease severity and could assist in the early identification of clinical deterioration (i.e., worsening physiological status, including hemodynamic instability, respiratory failure, and progression of organ dysfunction).

This study has several limitations. First, the single-center and retrospective design limits the generalizability of the findings to broader and more heterogeneous populations. The inclusion of only very elderly patients aged 80 years and older may restrict the applicability of the results to younger individuals with pneumonia. Furthermore, as all participants met ICU admission criteria, the study population may not fully represent the entire spectrum of older adults. Comorbidities were evaluated based solely on their number; validated scoring systems such as the Charlson Comorbidity Index (CCI), which could provide a more accurate assessment of their impact on mortality, were not used. Due to the retrospective nature of the study, detailed diagnostic data required for calculating the CCI were unavailable. Future studies incorporating validated comorbidity indices could enhance the accuracy of prognostic assessments.

In addition, the NPAR cutoff value used in this study was derived from a limited sample within a single center and demonstrated only moderate discriminatory performance (AUC: 0.692). Therefore, large-scale, multicenter, prospective validation studies are needed to assess the reliability and clinical applicability of this cutoff value. Although NPAR showed high sensitivity, its relatively low specificity suggests that it may not be sufficient as a standalone predictor of mortality and should be interpreted in conjunction with other clinical parameters.

Moreover, several potential confounding variables could not be evaluated due to data limitations. These include patients’ objective nutritional status, timing of antibiotic initiation, timing of ICU admission, characteristics of the causative pathogens (e.g., Gram-positive/negative bacteria, viruses, fungi), and the appropriateness and duration of antibiotic therapy. These limitations, inherent to the retrospective design, should be considered when interpreting the study findings.

## 5. Conclusions

The findings of this study suggest that the neutrophil percentage-to-albumin ratio (NPAR) may serve as a prognostic biomarker in pneumonia patients aged 80 years and older admitted to the intensive care unit. Elevated NPAR levels were significantly associated with increased disease severity, higher mortality, and a greater need for invasive mechanical ventilation. Cox regression analysis identified NPAR as an independent predictor of mortality, supporting its potential for clinical use. Given that it is an easily calculable and widely accessible parameter, NPAR may be a useful adjunct biomarker in predicting mortality and guiding clinical decision-making in elderly patients with pneumonia. However, large-scale, multicenter, prospective studies are needed to validate these findings and further assess the role of NPAR in clinical practice.

## Figures and Tables

**Table 1:** Comparison of Clinical and Laboratory Parameters Between Survivors and Non-survivors.

	Survivors (N = 82, 60.8%)	Non-Survivors (N = 53, 39.2%)	*p*-Value
Age, years (Mean ± SD)	86.37 ± 4.90	87.64 ± 4.97	0.463
Female Sex	38 (57.6%)	28 (42.4%)	0.163
Male Sex	44 (63.8%)	25 (36.2%)	
SOFA Score	5.59 ± 2.57	8.79 ± 2.56	<0.001
APACHE-II Score	19.35 ± 5.71	28.21 ± 3.73	<0.001
Need for Hemodialysis	9 (10.9%)	24 (89.1%)	<0.001
Need for Vasopressor Support	11 (12.1%)	32 (87.9%)	<0.001
Need for Invasive Mechanical Ventilation	12 (14.6%)	48 (85.4%)	<0.001
Presence of Comorbidities	63 (76.8%)	48 (90.6%)	0.042
COPD *	22 (26.8%)	24 (45.3%)	0.028
Malignancy	3 (3.7%)	7 (13.2%)	0.039
Neutrophil Percentage (%)	85 (78–90)	89 (85–92)	0.004
Albumin (g/L)	32 (27–35)	29 (24–34)	0.006
NPAR **	2.91 (2.44–21.60)	4.22 (2.98–30.92)	<0.001
Procalcitonin (ng/mL)	0.19 (0.06–0.67)	0.46 (0.21–2.63)	0.003
Lactate (mmol/L)	2.30 (1.40–3.00)	2.90 (2.40–3.40)	<0.001
Blood Urea Nitrogen (mg/dL)	51 (34–75)	82 (54–108)	<0.001
Creatinine (mg/dL)	1.13 (0.89–1.76)	1.52 (1.18–2.10)	0.010
Potassium (mmol/L)	4.00 (3.70–5.00)	4.49 (4.00–4.88)	0.102
CRP (mg/L)	32 (8–148)	62 (10–136)	0.456
Platelets (×10^3^/µL)	223 (181–293)	169 (144–202)	<0.001

* COPD: Chronic Obstructive Pulmonary Disease ** NPAR: Neutrophil Percentage-to-Albumin Ratio.

**Table 2:** Clinical Characteristics of Patients According to Neutrophil Percentage-to-Albumin Ratio Levels.

Variable	All Patients 135 (100%) N (%) Mean ± SD	NPAR ≤ 0.286 48 (35.6%) N (%) Mean ± SD *	NPAR > 0.286 87 (64.4%) N (%) Mean ± SD	*p*-Value
Age (years)	86.87 ± 4.95	86.65 ± 5.23	86.99 ± 4.82	0.638
Male sex	66 (48.9%)	22 (33.3%)	44 (66.7%)	0.599
SOFA score	6.84 ± 3.00	5.85 ± 2.94	7.39 ± 2.90	0.002
APACHE II score	22.83 ± 6.63	20.90 ± 7.53	23.90 ± 5.85	0.007
Renal replacement therapy	33 (24.4%)	10 (20.8%)	23 (26.4%)	0.470
Vasopressor requirement	43 (31.9%)	10 (20.8%)	33 (37.9%)	0.042
Invasive mechanical ventilation	60 (44.4%)	13 (27.1%)	47 (54.0%)	0.003
Requirement for high-flow nasal oxygen	82 (60.7%)	26 (54.1%)	56 (64.3%)	0.102
Requirement for noninvasive mechanical ventilation	68 (50.3%)	17 (35.4%)	51 (58.6%)	0.090
Presence of comorbidities	111 (82.2%)	47 (97.9%)	64 (73.6%)	<0.001
Cardiovascular disease	50 (37.0%)	25 (52.1%)	25 (28.7%)	0.007
Hypertension	67 (49.6%)	34 (70.8%)	33 (37.9%)	<0.001
Severity of the disease				
No sepsis	41 (30.4%)	20 (41.7%)	21 (24.1%)	0.035
Sepsis	94 (69.6%)	28 (58.3%)	66 (75.9%)	
SpO_2_/FiO_2_				
SpO_2_/FiO_2_ > 315	21 (15.6%)	6 (12.5%)	15 (17.2%)	0.087
235 < SpO_2_/FiO_2_ ≤ 315	43 (31.9%)	21 (43.8%)	22 (25.3%)	
148 < SpO_2_/FiO_2_ ≤ 235	42 (31.8%)	17 (35.4%)	25 (28.7%)	
SpO_2_/FiO_2_ ≤ 148	29 (21.5%)	4 (8.3%)	25 (28.7%)	
Mortality	53 (39.3%)	9 (18.8%)	44 (50.6%)	<0.001

* NPAR: Neutrophil Percentage-to-Albumin Ratio.

**Table 3:** Laboratory Findings of Patients According to Neutrophil Percentage-to-Albumin Ratio (NPAR).

Laboratory Parameters	All Patients 135 (100%) Median (IQR)	NPAR ≤ 0.286 48 (35.6%) Median (IQR)	NPAR > 0.286 87 (64.4%) Median (IQR)	*p*-Value
Neutrophil percentage (%)	86.8 (79.6–91.8)	84.90 (75.52–90.35)	88.20 (81.40–92.20)	0.008
Albumin (g/L)	30 (36–35)	34 (32–37)	28 (25–31)	<0.001
NPAR *	0.32 (0.25–2.58)	0.24 (0.22–0.26)	2.27 (0.33–2.89)	<0.001
Procalcitonin (ng/mL)	0.30 (0.12–0.92)	0.18 (0.05–0.59)	0.36 (0.15–1.59)	0.020
Lactate	2.60 (1.62–3.10)	2.35 (1.45–2.75)	2.90 (1.90–3.30)	0.003
Blood urea nitrogen (mg/dL)	60 (36–90)	58 (33–88)	65 (48–97)	0.167
Creatinine (mg/dL)	1.29 (1.0–1.95)	1.20 (0.90–1.90)	1.34 (1.06–1.97)	0.206
Potassium (mmol/L)	4.30 (3.89–4.90)	4.02 (3.70–4.80)	4.60 (4.10–5.08)	**<0.001**
C-reactive protein (CRP) (mg/L)	51 (9.4–141)	23 (4.9–141)	76 (39–142)	**0.011**
Neutrophil (×10^3^/µL)	10.20 (7.57–14.30)	9.58 (7.74–13.04)	10.80 (7.50–15.50)	0.176
Platelet count (×10^3^/µL)	197 (163–247)	200 (160–251)	193 (164–242)	0.811

* NPAR: Neutrophil Percentage-to-Albumin Ratio.

**Table 4:** ROC Analysis Results of Neutrophil Percentage-to-Albumin Ratio (NPAR) for Predicting Mortality.

	AUC	95% Confidence Interval	Cut-Off Value	Sensitivity (%)	Specificity (%)	PPV (%)	NPV (%)	LR+ *	LR− **	*p*-Value
NPAR ***	0.692	0.599–0.784	0.286	83	47.6	50.6	81.2	1.58	0.36	<0.001

* LR+: Positive likelihood ratio; ** LR−: Negative likelihood ratio *** NPAR: Neutrophil Percentage-to-Albumin Ratio.

**Table 5:** Cox Regression Analysis Results for Factors Associated with Mortality.

Variable	Univariate Cox Regression		Multivariate Cox Regression	
	HR (95% CI)	*p*-Value	HR (95% CI)	*p*-Value
Age	1.045 (0.989–1.104)	0.114		
Presence of comorbidities	2.290 (0.911–5.760)	0.078		
Cardiovascular disease	0.830 (0.469–1.471)	0.524		
Hypertension	0.682 (0.394–1.180)	0.172		
APACHE II score	1.163 (1.118–1.209)	**<0.001**	1.077 (1.013–1.147)	**0.019**
SOFA score	1.291 (1.193–1.398)	**<0.001**	1.100 (0.964–1.254)	0.156
NPAR * > 0.286	3.318 (1.616–6.812)	**0.001**	2.488 (1.167–5.302)	**0.018**
Need for renal replacement therapy	3.788 (2.185–6.567)	**<0.001**	1.969 (1.046–3.705)	**0.036**
Need for vasopressor therapy	4.166 (2.385–7.279)	**<0.001**	0.616 (0.311–1.220)	0.165
Need for invasive mechanical ventilation	20.297 (8.019–51.374)	**<0.001**	9.446 (3.402–26.229)	**<0.001**

* NPAR: Neutrophil Percentage-to-Albumin Ratio.

## Data Availability

The original contributions presented in this study are included in the article. Further inquiries can be directed to the corresponding author.
